# Reliance on topographical coding alone may overestimate survival in pancreatic ductal adenocarcinoma

**DOI:** 10.1016/j.sipas.2026.100357

**Published:** 2026-07-28

**Authors:** William Falk, Roland Andersson, Daniel Ansari

**Affiliations:** Department of Surgery, Clinical Sciences Lund, Lund University, Skåne University Hospital, Lund, Sweden

**Keywords:** C25, ICD-O-3 topography code, ICD-O-3 morphology code, Pancreatic cancer, Pancreatic ductal adenocarcinoma, Survival

## Abstract

**Background:**

In epidemiological and outcomes research, “pancreatic cancer” is frequently used interchangeably with pancreatic ductal adenocarcinoma (PDAC), the most common and most lethal pancreatic cancer subtype. However, relying solely on an anatomic site code may capture a mix of pancreatic tumor types with better prognoses than PDAC, leading to inflated survival estimates.

**Methods:**

We used the Surveillance, Epidemiology, and End Results (SEER) registry to compare data derived from site-only case ascertainment (C25.x, excluding C25.4) versus histologically confirmed PDAC using ICD-O-3 morphology codes. Only microscopically confirmed cases were included. We quantified differences in patient demographics, tumor characteristics, and overall survival between the two case-selection strategies. Survival estimates were generated using Kaplan-Meier methods and multivariable Cox regression models.

**Results:**

A total of 283,465 patients with C25 tumors (excluding C25.4) were identified. Based on morphology coding, 233,421 (82.3%) tumors were classified as PDAC and 50,044 (17.7%) as non-PDAC histology. The 5-year survival rate in the C25 cohort (excluding C25.4) was 12.1%, whereas survival among PDAC patients alone was 6.0%. For surgically resected patients, the 5-year survival rate in the C25 cohort (excluding C25.4) was 35.3%, compared to 21.0% for those with PDAC histology. In multivariable Cox regression analysis, PDAC histology was significantly associated with reduced overall survival (HR 2.57, 95% CI 2.54–2–61, p < 0.001).

**Conclusion:**

Case definitions based on the C25 site code alone lead to overestimation of true PDAC survival. Histology-based selection is necessary for accurate research on PDAC, underscoring the importance of precise tumor classification in registry-based research.

## Introduction

Pancreatic cancer is currently one of the most lethal malignancies in the United States and globally, with mortality rates closely mirroring incidence due to its aggressive biology and the absence of early symptoms [1,2]. Pancreatic ductal adenocarcinoma (PDAC) accounts for approximately 80–90% of all pancreatic cancers and therefore dominates both clinical management and epidemiologic characterization of the disease [3,4]. For this reason, PDAC is often used synonymously with “pancreatic cancer” in the scientific literature [3,[5], [6], [7], [8]]. While this may simplify communication, it can also obscure important heterogeneity among pancreatic tumor subtypes that differ substantially in natural history, treatment responsiveness, and survival outcomes [[9], [10], [11]].

Cancer registries serve as a cornerstone of pancreatic cancer research. They provide population-level data that support epidemiologic studies, and the identification of biomarkers when integrated with biomedical datasets. In registry-based research, pancreatic tumors are often identified using the ICD-O-3 topography code C25, which captures all neoplasms arising within the pancreas regardless of histology [[12], [13], [14], [15]]. Although this strategy ensures complete anatomic coverage, reliance on anatomic site alone introduces the risk of misclassification. Several non-PDAC histologies, such as pancreatic neuroendocrine tumors (PNETs), acinar cell carcinoma, mucinous cystic neoplasms with invasive carcinoma, and solid pseudopapillary neoplasms, are also assigned to the C25 site code but have markedly better prognoses than PDAC [11,[16], [17], [18], [19]]. As a result, studies defining pancreatic cancer solely through C25 risk inflating survival estimates relative to true PDAC. This misclassification has important epidemiologic consequences [20]. Because survival for PDAC is extremely poor, with a 5-year survival rate close to 5% for histologically verified cases [4], even a small admixture of more indolent tumors can produce substantial bias. Such dilution may lead to erroneous conclusions regarding fundamental aspects of PDAC research, including diagnosis, prognosis, and treatment.

To address this gap, the present study uses a large population-based registry to compare case characteristics and survival outcomes between tumors identified by pancreatic anatomic site code (C25) and those defined explicitly by PDAC-consistent histology codes. By quantifying discrepancies between anatomic-only versus histologically confirmed PDAC case definitions, this analysis aims to evaluate the extent to which anatomic site codes alone misclassify pancreatic cancers and to determine the impact of such misclassification on survival estimates.

## Methods

### Data source

We conducted a retrospective cohort study using data from the Surveillance, Epidemiology, and End Results (SEER) program of the National Cancer Institute. SEER provides population-based cancer incidence and survival data covering approximately 45.9% of the U.S. population. Data were obtained from the SEER Research Database [21], which includes information on patient demographics, tumor characteristics, stage, treatment, and vital status. The study conformed to STROBE guidelines [22].

### Study population

We identified all primary malignant pancreatic tumors diagnosed between 2004 and 2022 using the ICD-O-3 topography codes: C25.0-C25.9 (excluding C25.4). To improve diagnostic accuracy, we restricted the cohort to microscopically confirmed tumors. Within this cohort, PDAC cases were identified using ICD-O-3 histology codes for PDAC and its related subtypes based on the World Health Organization (WHO) classification, including conventional PDAC (8140/3, 8500/3), colloid carcinoma (8480/3), poorly cohesive carcinoma (8490/3), medullary carcinoma NOS (8510/3), adenosquamous carcinoma (8560/3), hepatoid carcinoma (8576/3), large cell carcinoma with rhabdoid phenotype (8014/3), carcinoma, undifferentiated, NOS (8020/3), and undifferentiated carcinoma with osteoclast-like giant cells (8035/3) [4,23]. All remaining C25 cases were categorized as non-PDAC pancreatic malignancies.

### Outcome

The primary outcome was overall survival, defined as the time from diagnosis to death from any cause or last follow-up, consistent with SEER methodology. Vital status and survival time were extracted directly from the SEER database.

### Statistical analysis

Baseline characteristics were compared between groups using chi-square tests for categorical variables and Wilcoxon rank-sum tests for continuous variables. Overall survival was estimated using the Kaplan–Meier method, and survival differences were evaluated with the log-rank test. Multivariable Cox proportional hazards regression models were constructed to estimate hazard ratios (HRs) and 95% confidence intervals for the association between histology category (PDAC vs. C25; PDAC vs. non-PDAC) and overall survival, adjusting for all prespecified covariates.

## Results

### Cohort characteristics

**Fig. 1:**
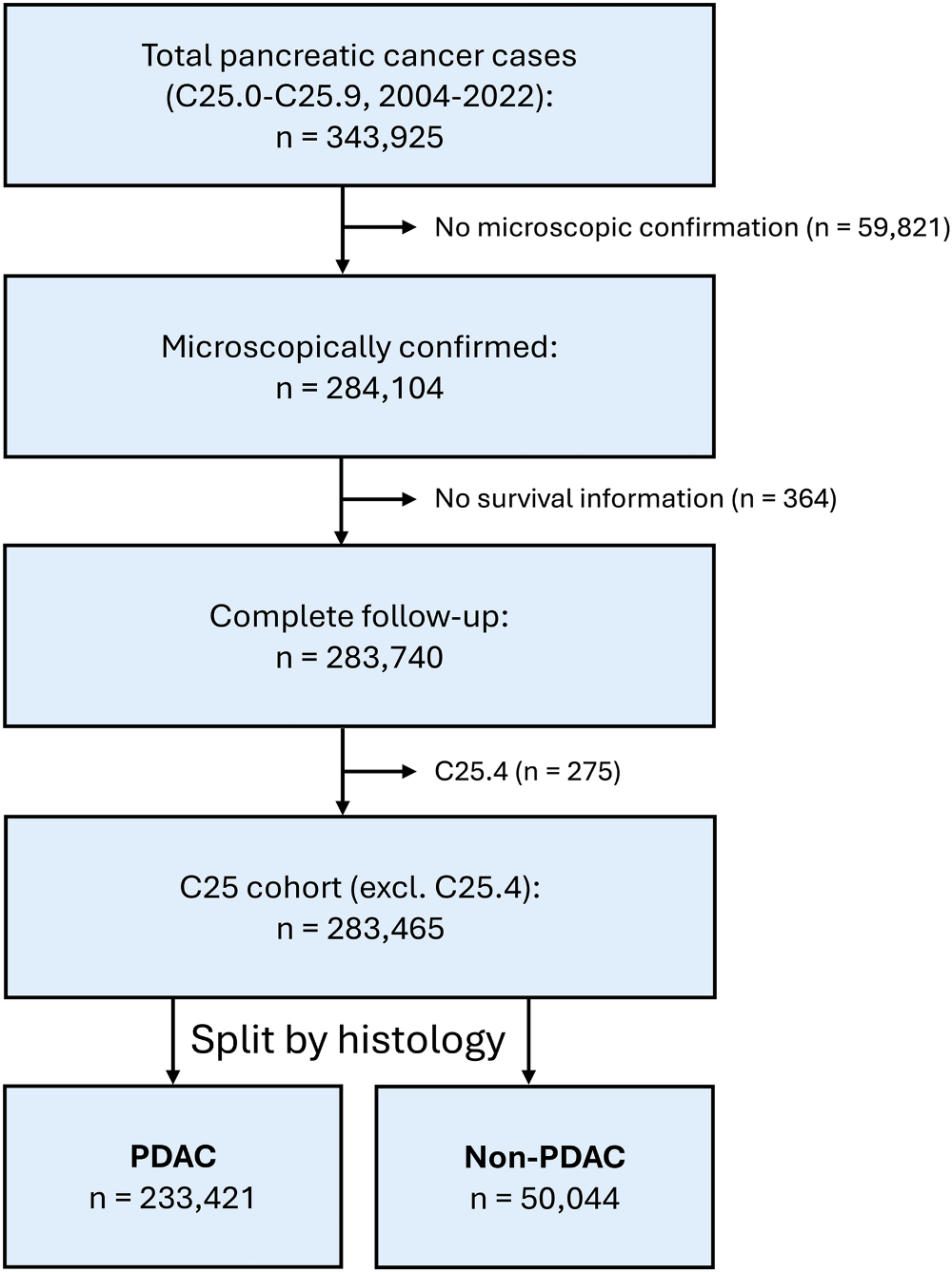
STROBE flow diagram.

From the SEER registry, we identified 343,925 patients diagnosed with C25 tumors during the study period. After excluding cases with C25.4 coding and missing histological and survival data, the final cohort consisted of 283,465 cases, of which 233,421 were classified pathologically as PDAC and 50,044 as non-PDAC histology (Fig. 1). Compared with the non-PDAC group, patients with PDAC were older and had a slightly higher proportion of females. Tumors located in the pancreatic head were more common in the PDAC group. Furthermore, PDAC patients were more likely to present with advanced-stage disease, and less likely to undergo surgical resection. Baseline characteristics are summarized in Table 1.

**Table 1:** Comparison of clinical characteristics between patients with PDAC and those with non-PDAC histology.

Variable	PDAC, N = 233,421	Non-PDAC histology, N = 50,044	P-value
Age, years	69 (61–77)	66 (56–75)	<0.001
Female sex	112,479 (48.2%)	23,539 (47.0%)	<0.001
Tumor location (head)	119,381 (51.1%)	16,755 (33.5%)	<0.001
Stage			<0.001
Localized	22,652 (9.7%)	13,983 (27.9%)	
Regional	79,438 (34.0%)	9218 (18.4%)	
Distant	123,114 (52.7%)	22,052 (44.1%)	
Unknown	8217 (3.5%)	4791 (9.6%)	
Surgical resection	46,757 (20.0%)	17,061 (34.1%)	<0.001
Chemotherapy	133,953 (57.4%)	14,007 (28.0%)	<0.001
Time era			<0.001
2004–2013 2014–2022	101,228 (43.4%) 132,193 (56.6%)	22,072 (44.1%) 27,972 (55.9%)	

### Histological findings in the non-PDAC group

**Fig. 2:**
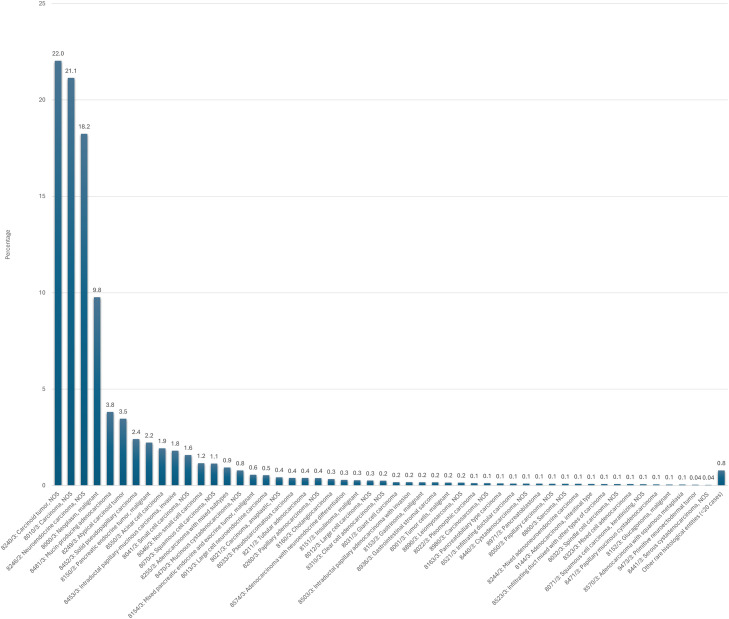
Distribution of histological findings in patients with non-PDAC histology (n = 50,044).

The non-PDAC subgroup exhibited heterogeneous histology, with PNETs being the most common entity (Fig. 2). This highlights that topographical coding alone (C25.4) was insufficient to accurately exclude PNETs, and without ICD-O-3 histology coding, a substantial number of tumors would have been misclassified as exocrine pancreatic cancers. Additionally, within the non-PDAC category, we identified non-pancreatic malignancies, such as cholangiocarcinomas, sarcomas, and metastatic lesions, further contributing to misclassification.

### Overall survival

**Fig. 3:**
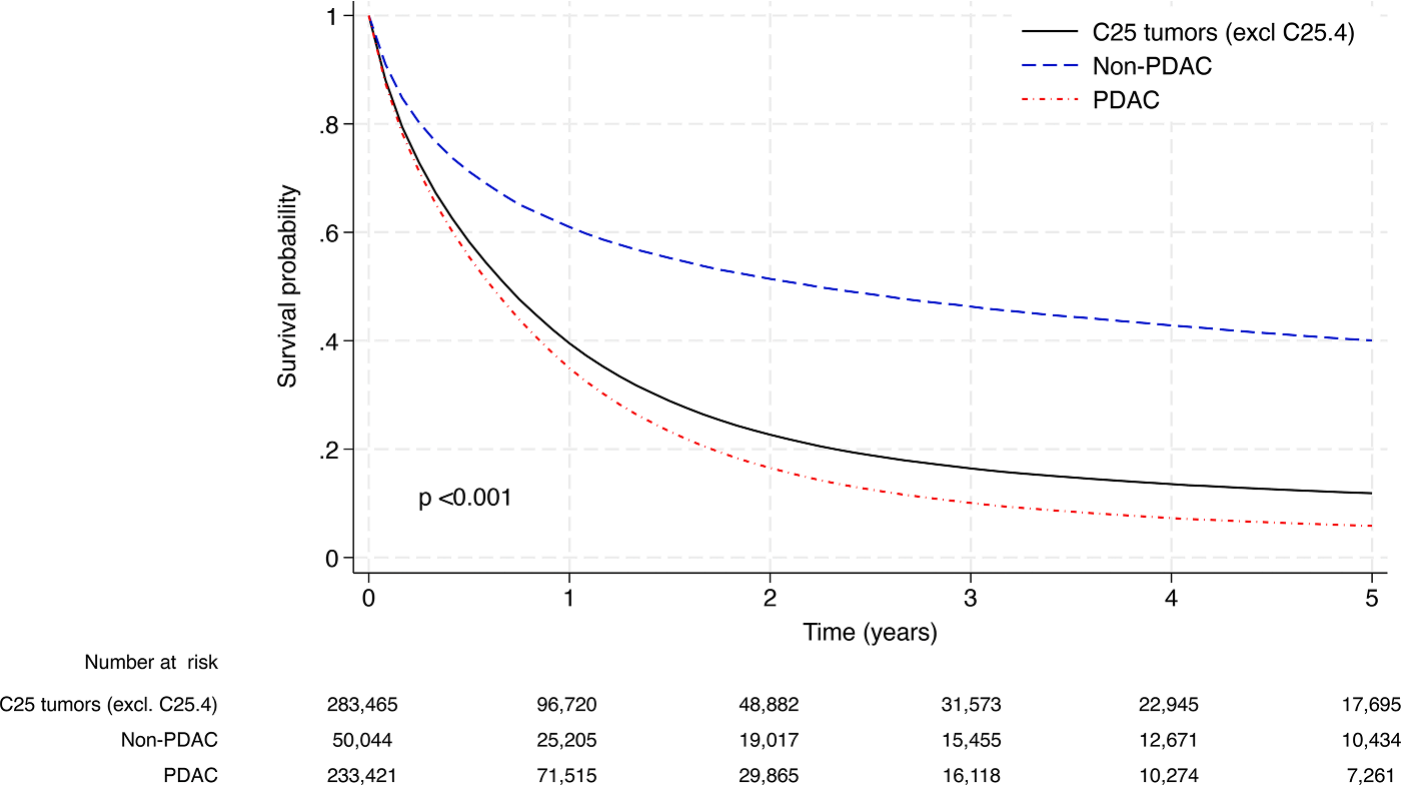
Overall survival for patients with C25 tumors (excl. C25.4) stratified by histological type (PDAC vs non-PDAC).

**Fig. 4:**
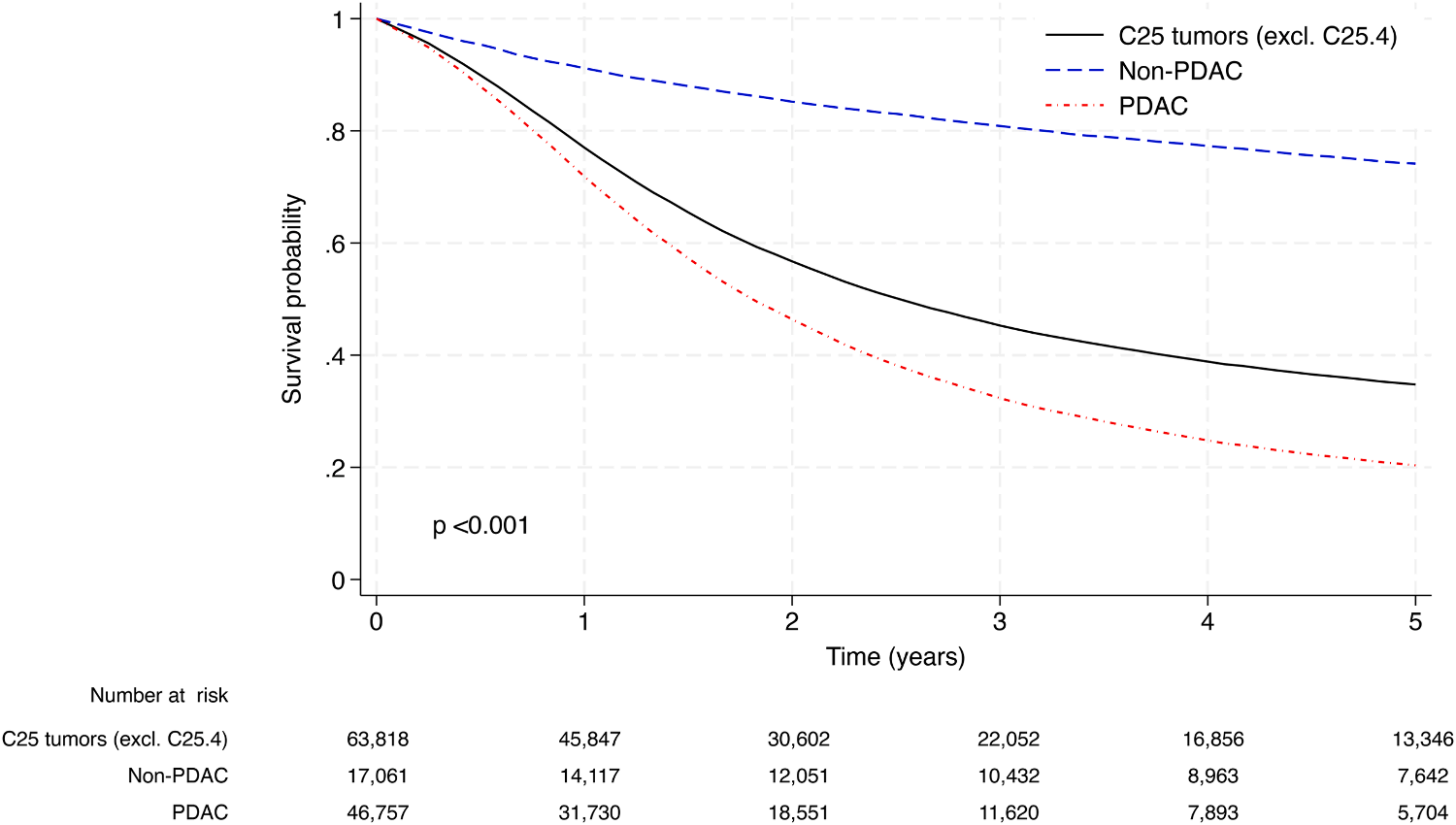
Overall survival for surgically resected patients with C25 tumors (excl. C25.4) stratified by histological type (PDAC vs non-PDAC).

In the overall cohort of patients with C25 tumors (excluding C25.4), median survival was 9 months with a 5-year survival rate of 12.1% (Fig. 3). Patients who underwent surgical resection had a median survival of 31 months and a 5-year survival rate of 35.3% (Fig. 4).

When the full cohort was stratified by tumor type, patients with PDAC demonstrated poor outcomes, with limited median (8 months) and 5-year survival (6.0%). In contrast, the non-PDAC group had significantly longer survival (median survival: 27 months, 5-year survival: 40.4%), p < 0.001. This more favorable non-PDAC subgroup drives an inflation of survival estimates when all C25 tumors are analyzed together. A similar pattern was observed when analyses were restricted to surgically resected cases. Also among resected patients, survival for PDAC remained poor (median survival: 22 months, 5-year survival: 21.0%), whereas non-PDAC tumors showed much better outcomes (median survival: 163 months, 5-year survival: 74.5%), p < 0.001. As a result, inclusion of non-PDAC tumors in combined analyses can substantially overestimate survival for “pancreatic cancer” unless PDAC is examined separately.

### Multivariable survival analysis

In a Cox proportional hazards model adjusted for age, sex, tumor location, stage, surgical resection, chemotherapy and time era, PDAC remained independently associated with significantly worse overall survival: PDAC vs non-PDAC histology: HR = 2.57, 95% CI 2.54–2.61, p < 0.001 (Table 2).

**Table 2:** Cox regression analysis for overall survival.

Variables	HR	95% CI	P-value
PDAC vs non-PDAC histology, Unadjusted	2.54	2.51–2.58	<0.001
PDAC vs non-PDAC histology, Adjusted for age and sex	2.31	2.28–2.34	<0.001
PDAC vs non-PDAC histology, Adjusted for 7 covariates*	2.57	2.54–2.61	<0.001

⁎ Age, sex, tumor location, stage, surgical resection, chemotherapy and time era.

### Sensitivity analysis

A sensitivity analysis was performed excluding histologies representing non-primary pancreatic malignancies, including hepatobiliary primaries, sarcomas, and metastatic tumor types identified by ICD-O-3 morphology codes. After exclusion of 542 cases with non-pancreatic histologies, the sensitivity cohort comprised 282,923 patients. In the fully adjusted Cox proportional hazards model, PDAC remained associated with inferior overall survival compared with non-PDAC tumors (HR = 2.59, 95% CI 2.55–2.63; p < 0.001), comparable to the estimate observed in the primary analysis.

## Discussion

In this population-based analysis using the SEER registry, we compared overall survival among tumors classified by anatomical site code C25 (excluding C25.4) with survival of cases identified specifically by PDAC histological codes. Our findings demonstrate that C25-based cohorts significantly overestimate survival relative to true PDAC, underscoring a critical methodological issue in pancreatic cancer research.

Because PDAC accounts for the majority of pancreatic cancer diagnoses, researchers may limit themselves to site codes when histology is unavailable or considered redundant. However, as we have shown in this study, the C25 code encompasses a heterogeneous mix of pancreatic tumors (Fig. 2), such as PNETs, mucinous cystic neoplasms, solid pseudopapillary carcinoma, acinar cell carcinoma, and invasive IPMNs, entities that frequently have substantially better prognoses than PDAC. Mixing these histologies within a single C25-defined cohort may lead to inflation of survival estimates, creating the impression that PDAC has a more favorable prognosis than it actually does.

O’Rorke et al. [20] recently conducted an audit of published UK Biobank studies relating to PDAC or exocrine pancreatic tumors. Half of these studies (5/10) only used topographical coding without utilization of histological coding. This may have led to the inadvertent inclusion of PNETs and other non-exocrine tumors labeled as pancreatic cancer. Bhuller et al. [24] performed a quality‑control registry study showing substantial misclassification when using site code data alone. Many cases labeled as “pancreatic cancer” were actually PNETs or other non‑exocrine malignancies, or lacked histological verification. This mirrors the problem described in the UK Biobank context. In another study, Hwang et al. [25] validated how well topographical coding in a claims database matches pathologically confirmed pancreatic cancer. It was found that a portion of cases clinically coded as “pancreatic cancer” represented cystic neoplasms, non-exocrine tumors or metastases, further highlighting the limitations of relying solely on site-based coding. Arous et al. [26] combined data derived from a hospital‑information system and a surgical database and noted that a substantial fraction of patients identified by ICD‑codes alone lacked histologically confirmed pancreatic neoplasms, again underscoring problems of non‑morphology coding.

These findings have several important implications. Epidemiological and outcomes studies that rely on site codes alone may generate biased estimates of survival, treatment patterns, or mortality trends. Clinical effectiveness studies in particular risk misclassification that may dilute or misrepresent therapeutic benefit. From a public health perspective, reports based on C25 may convey overly optimistic expectations regarding pancreatic cancer outcomes, potentially affecting advocacy, funding priorities, and patient counseling. Our results argue for the routine incorporation of both site and histology codes when identifying PDAC within registry-based datasets.

Several limitations warrant consideration. First, SEER registry data are subject to misclassification errors, including potential inaccuracies in histologic coding. Although SEER’s coding standards are robust, subtle differences in diagnostic practices across institutions may influence histologic categorization. Second, detailed treatment information, such as chemotherapy regimens [27], surgical margins, or molecular profiling, is limited in SEER, preventing adjustment for treatment-level confounders. Third, temporal changes in diagnostic imaging, pathology classification, and coding practices may affect comparisons across years. Nonetheless, the magnitude and consistency of the observed survival differences suggest that the primary findings are less likely to be attributable to these limitations.

## Conclusions

This study demonstrates that using ICD site code C25 (excluding C25.4) as a proxy for PDAC substantially overestimates survival, due to the inclusion of less aggressive pancreatic tumor subtypes. These findings highlight the importance of using combined site- and histology-based definitions in epidemiological and outcomes research. Improved precision in case identification will yield more accurate survival estimates, more valid comparative analyses, and better alignment of public health reporting with the true clinical behavior of PDAC.

## Ethics approval and consent to participate

The SEER registry provides patient-level data that were fully anonymized prior to our analysis. The Swedish Ethical Review Authority approved the study protocol (record number 2024–02,360-01). The need for informed consent was waived by the ethics committee.

## Funding

This work was supported by the Swedish Cancer Society, the Swedish Research Council, the Swedish Surgical Society, the Crafoord Foundation, the Ingrid and Sverker Persson Foundation, Regional Research Support and ALF funding from Region Skåne.

## Availability of data and materials

The datasets analyzed during the current study are available from the corresponding author on reasonable request.

## Ethics declaration

### Informed consent and patient details

The authors have NOT obtained informed consent from participants or their legal representatives. The SEER registry provides patient-level data that were fully anonymized prior to our analysis. The Swedish Ethical Review Authority approved the study protocol (record number 2024–02,360-01). The need for informed consent was waived by the ethics committee.

### Studies in human

This study was performed in compliance with relevant laws, regulatory frameworks and guidelines where the research took place.

This study was approved by the Swedish Ethical Review Authority.

(Approval No. 2024–02,360-01)

## CRediT authorship contribution statement

**William Falk:** Writing – original draft, Data curation. **Roland Andersson:** Writing – review & editing, Supervision. **Daniel Ansari:** Writing – review & editing, Supervision, Methodology, Funding acquisition, Conceptualization.

## Declaration of competing interest

The authors declare that they have no known competing financial interests or personal relationships that could have appeared to influence the work reported in this paper.
